# Courtship behavior, nesting microhabitat, and assortative mating in sympatric stickleback species pairs

**DOI:** 10.1002/ece3.7164

**Published:** 2021-01-29

**Authors:** Laura L. Dean, Hannah R. Dunstan, Amelia Reddish, Andrew D. C. MacColl

**Affiliations:** ^1^ School of Life Sciences University of Nottingham Nottingham UK

**Keywords:** assortative mating, behavior, courtship, evolution, gene flow, mate choice

## Abstract

The maintenance of reproductive isolation in the face of gene flow is a particularly contentious topic, but differences in reproductive behavior may provide the key to explaining this phenomenon. However, we do not yet fully understand how behavior contributes to maintaining species boundaries. How important are behavioral differences during reproduction? To what extent does assortative mating maintain reproductive isolation in recently diverged populations and how important are “magic traits”? Assortative mating can arise as a by‐product of accumulated differences between divergent populations as well as an adaptive response to contact between those populations, but this is often overlooked. Here we address these questions using recently described species pairs of three‐spined stickleback (*Gasterosteus aculeatus*), from two separate locations and a phenotypically intermediate allopatric population on the island of North Uist, Scottish Western Isles. We identified stark differences in the preferred nesting substrate and courtship behavior of species pair males. We showed that all males selectively court females of their own ecotype and all females prefer males of the same ecotype, regardless of whether they are from species pairs or allopatric populations. We also showed that mate choice does not appear to be driven by body size differences (a potential “magic trait”). By explicitly comparing the strength of these mating preferences between species pairs and single‐ecotype locations, we were able to show that present levels of assortative mating due to direct mate choice are likely a by‐product of other adaptations between ecotypes, and not subject to obvious selection in species pairs. Our results suggest that ecological divergence in mating characteristics, particularly nesting microhabitat may be more important than direct mate choice in maintaining reproductive isolation in stickleback species pairs.

## Graphical abstract

What is the role of behavior in speciation? We identify a number of key behavioural differences that contribute to reproductive isolation in a model system, the three‐spined stickleback (*Gasterosteus aculeatus*) species complex. However, we show that assortative mating is present regardless of contact between ecotypes, and thus, direct selection on mating behavior in regions of secondary contact may not play an important role.

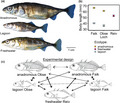

## 1 INTRODUCTION

Behavior dictates the way in which an organism interacts with other members of the same species, with other living entities, and with its surrounding environment (Levitis et al., 2009). It is fundamental for survival and reproduction and is particularly interesting in the light of evolution, because differences in reproductive behavior can play a crucial role in advancing, maintaining, and breaking down boundaries between species. Gene flow between divergent populations can lead to hybridization and homogenization of two populations, but population‐level differences and the progression of speciation can also be maintained in the face of gene flow. The latter has been a particularly contentious topic for decades (Berlocher & Feder, 2002; Bird et al., 2012; Bolnick, 2011; Bolnick & Fitzpatrick, 2007; Dieckmann & Doebeli, 1999; Smith, 1966; Smith et al., 2013; Via, 2001), but today, it is accepted that, under certain circumstances, new species can emerge while exchanging genes (Foote, 2018; Papadopulos et al., 2019; Richards et al., 2019). Divergent mating behavior and active mate choice provide a potential mechanism for this phenomenon (Kirkpatrick, 2001; Liu et al., 2011), particularly with regard to reproductive isolation between recently diverged populations because behavioral differences can arise quickly, whereas postzygotic barriers to gene flow (i.e., hybrid sterility or inviability) tend (although not always) to require a suite of mutations which take longer to accumulate (Coyne & Orr, 1989, 1997; Rull et al., 2013). However, our understanding of how reproductive behavioral differences arise and how important they are for reproductive isolation in wild populations is incomplete.

Behavioral differences underlie most prezygotic isolation, either directly via active mate choice, or indirectly via temporal or ecological differences during reproduction. Divergent mating behavior can theoretically even complete the speciation process in the face of gene flow (Dobzhansky, 1937; Rice & Hostert, 1993; Servedio & Noor, 2003). Classically, these differences in mating behavior are thought to evolve as a result of direct selection for assortative mating or via reinforcement (selection against the production of hybrids of reduced fitness) (Pfennig, 2016). However, they can also arise as a by‐product of other adaptations without any selection for assortative mating, for example, one population might evolve reduced courtship displays to avoid predation while another might solve the same problem by nesting/courting in dense foliage, leading to reduced cross‐population mating as a by‐product of other adaptations, a phenomenon which is often overlooked (Rice & Hostert, 1993; Vines & Schluter, 2006). Low‐level gene flow between divergent populations with incomplete postzygotic barriers to hybridization is common in nature (Campagna et al., 2014; Moritz et al., 2009; Ravinet et al., 2013; Sa‐Pinto et al., 2010), but we know far less than we should about the prevalence and role of divergent mating behavior in these populations.

Contact between phenotypically and ecologically divergent ecotypes with varying degrees of reproductive isolation occurs throughout the Holarctic range of the three‐spined stickleback (*Gasterosteus aculeatus*, hereafter “stickleback”), largely as a result of their early Holocene marine to freshwater radiation (Bell et al., 2004; Jones et al., 2012; Magalhaes et al., 2016; Taylor & McPhail, 2000). Stickleback also perform a suite of well‐characterized courtship behaviors (Candolin, 1997; Hughes et al., 2013; Tinbergen, 1952), making the stickleback radiation an ideal system for investigating the role of mating behavior in maintaining species boundaries in the face of gene flow. Assortative mating (the ability to recognize and choose to mate with conspecific individuals) is common between reproductively isolated stickleback ecotypes and has been documented in benthic–limnetic (Bay et al., 2017; Kozak et al., 2011), lake–stream (Andreou et al., 2017), lava–nitella (Olafsdottir et al., 2006), and anadromous–freshwater resident (Furin et al., 2012) species pairs. This assortative mating can be driven by variation in factors directly involved in mating interactions such as mating behavior (Ishikawa & Mori, 2000), body size (McKinnon et al., 2012), nuptial coloration (McKinnon, 1995), and nest structure (Blouw & Hagen, 1990), or variation in spatial and temporal aspects of courtship that result in fine‐scale segregation of phenotypes (Borzee et al., 2016; Hagen, 1967; Pegoraro et al., 2016; Snowberg & Bolnick, 2012). Divergence in body size is a particularly important aspect of almost all stickleback species pairs and body size has been implicated as a potential “magic trait” in this system, involved in both ecological adaptation and assortative mating (Bay et al., 2017; Conte & Schluter, 2013; Head et al., 2013; MacColl, 2009; McKinnon et al., 2004; Nagel & Schluter, 1998; Schluter, 1993). However, the role of mating behavior in maintaining reproductive isolation and the frequency with which assortative mating arises because it is itself either directly or indirectly (e.g., through reinforcement) selected for remains uncertain (Bolnick & Kirkpatrick, 2012; Vines & Schluter, 2006).

**FIGURE 1:**
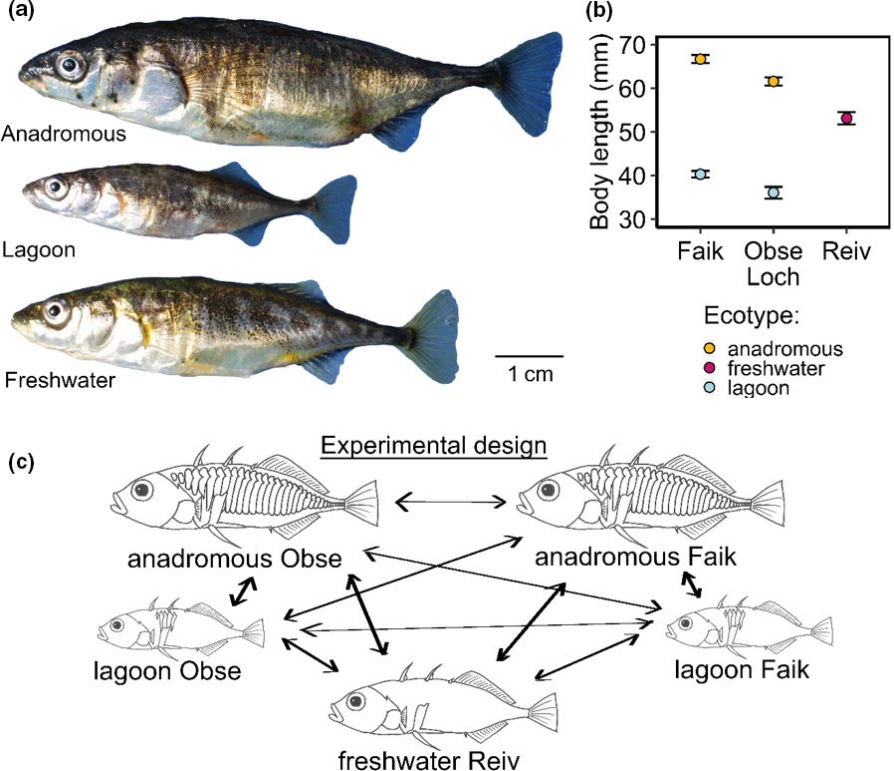
Ecotype characteristics and experimental design. (a) Photographs showing examples of (female) anadromous and lagoon fish from Obse and freshwater fish from Reiv. (b) Mean body length for each ecotype from each lake. Means are calculated using all fish successfully used in mate choice trials. (c) Diagram showing the experimental design: all possible trials between different ecotypes from different lochs were conducted. Arrows indicate pairings for mate choice trials

The island of North Uist, Scottish Western Isles is covered by a mosaic of interconnected freshwater and brackish lochs and coastal lagoons, most of which have been colonized by stickleback since the last glacial retreat 10,000–20,000 YBP (Ballantyne, 2010). Stickleback populations on North Uist vary extensively in morphological and associated genetic characteristics (MacColl et al., 2013; Magalhaes et al., 2016). The island contains isolated allopatric ecotypes and genetically and phenotypically distinct species pairs. Anadromous stickleback migrate from the open ocean into coastal lagoons and streams during the spring breeding season, during which they breed sympatrically alongside lagoon resident (hereafter “lagoon”) and freshwater resident (hereafter “freshwater”) ecotypes that do not migrate to sea. Anadromous fish are much larger, more heavily armored and differ from resident ecotypes in body shape and various trophic morphological traits such as gill raker number, reflecting their more pelagic lifestyle (see Figure 1 for photographs of different ecotypes). Reproductive isolation in species pairs is strong despite low levels of gene flow (Dean et al., 2019), and there is likely strong selection against hybrids, many of which probably attempt to migrate to sea without the full suite of associated traits. Hybrid fish, for example, often exhibit an intermediate lateral plate phenotype (Dean et al., 2019), which would likely make them more vulnerable to predation. Assortative mating may be important for maintaining reproductive isolation in lagoon–anadromous species pairs but, to the best of our knowledge, has never previously been tested.

Here we focus on investigating the behavioral mechanisms responsible for maintaining reproductive isolation in recently described lagoon–anadromous species pairs. We use no‐choice mating trials to investigate ecotype‐level differences in male nesting and courtship behavior and female mate choice. Evidence that reproductively isolated ecotypes both display positive assortative mating and differ in nesting habits and courtship behavior would suggest that these behavioral differences may be responsible for maintaining reproductive isolation in the face of gene flow. To investigate whether the sympatric condition of species pairs is responsible for assortative mating, we also compare levels of assortative mating in two sympatric species pair populations with those in a naïve landlocked allopatric population (as a best available proxy for allopatric populations of the same ecotypes, as species pair ecotypes do not occur allopatrically). A preference for a corresponding ecotype in fish from an allopatric population can only have arisen as a by‐product of other adaptations and not from either direct selection on mating preferences or reinforcement (as there is no contact between allopatric ecotypes). We, therefore, predict that if it is specifically selected for assortative mating between sympatric ecotypes will likely be stronger than that of the allopatric population. Taken together, our findings shed light on the role of behavior in adaptive divergence and in maintaining reproductive isolation in sympatry.

## 2 MATERIALS AND METHODS

### 2.1 Fish collection and husbandry

In April–May 2016 and 2017, stickleback were caught from three lochs on North Uist: two that contain sympatric species pairs of lagoon and anadromous fish (Obse and Faik), and a third, containing an isolated, solitary, allopatric population (Reiv, see Table 1 for detailed loch information) using unbaited minnow traps (Gee traps, Dynamic Aqua, Vancouver) set overnight in water 30‐100 cm deep. For use in mate choice trials, fish in breeding condition (males displaying full nuptial coloration and heavily gravid females) were selected from each catch and transported to a rental property on the island in aerated loch water, after which they were transferred to loch specific stock tanks containing either freshwater (dechlorinated tap water) or ~20‐30 ppt brackish water, depending on the salinity of source lochs (Table 1), and some weed collected from source lochs for cover. Brackish water was either pumped directly from the sea and mixed with dechlorinated tap water to achieve the required salinity or prepared using Seamix artificial sea water mix and dechlorinated tap water. All fish were fed on washed, defrosted bloodworm once a day and were kept in stock tanks until required for mate choice trials, after which they were anaesthetized using an overdose of MS222 and killed in accordance with Schedule 1 of UK Home Office regulations for use in other research.

**TABLE 1:** Stickleback sampling sites

Loch name	ID	*n*	Salinity	Location
Ob nan Stearnain	Obse	30 (50) [0]	brackish	57°36′6′′N; 7°10′22′′W
Fairy Knoll	Faik	34 (35) [0]	brackish	57°38′7′′N; 7°12′54′′W
na Reivil	Reiv	0 (0) [33]	freshwater	57°36′39′′N; 7°30′50′′W

Note

Sample sizes for mate choice trials are given for lagoon fish, anadromous fish (curved parentheses), and freshwater fish (square parentheses). Brackish salinity classifications describe water with absolute conductivity 20,000–35,000 µS/cm and freshwater: absolute conductivity < 500 µS/cm. Sampling locations are given in latitude, followed by longitude.

### 2.2 Mate choice trials

To investigate ecotype‐level differences in male nesting and courtship behavior and female mate choice, female no‐choice mating trials were conducted between the lagoon and anadromous ecotypes from the two species pairs (Obse and Faik). Ideally, to determine whether sympatry (and therefore, contact) with other stickleback ecotypes drives assortative mating, comparisons of the strength of assortative mating would be made between sympatric ecotypes and morphologically and ecologically similar, but solitary, allopatric ecotypes. However, there are no known locations on North Uist in which anadromous or lagoon ecotypes (which both occur in brackish water) spawn allopatrically. As a best available proxy, an allopatrically breeding freshwater population (Reiv), that was morphologically as intermediate as possible between lagoon and anadromous ecotypes (Figure 1a, b), was selected for comparison. Our experimental design included trials involving all possible pairings between the three different ecotypes (anadromous, lagoon, and freshwater) from three different populations (Obse, Faik, and Reiv), Figure 1c. This allowed for testing the effects of ecotype and loch (within species pair trials) simultaneously.

No‐choice, as opposed to choice, trials were used as they generally work well in stickleback, providing a more conservative estimate of mating preferences than choice trials (Coyne, 1993; Dougherty & Shuker, 2015; Furin et al., 2012). Furthermore, pilot studies showed that lagoon males did not construct nests in the presence of anadromous males and were frequently bullied when males of both ecotypes were housed together. Nest destruction between competing ecotypes is also common in a confined environment (Nagel & Schluter, 1998), making choice trials impractical.

Trials were conducted outside so as to maintain consistent temperature and lighting conditions, in 55 L clear plastic boxes filled with fresh (for freshwater males) or brackish water (for lagoon and anadromous males) prepared as in stock tanks. Each box contained at least one rock for cover, some aquatic plant material collected from stickleback source lagoons, 200 seven centimeter long black cotton threads, which could be used as nesting material (Smith & Wootton, 1999) (but in fact were not utilized by males in our experiments), and two large petri dishes, one filled with sand, and one with gravel collected from nearby lochs, for nesting substrate. Brackish (lagoon and anadromous) males were also given an additional large petri dish containing mud, and a seaweed covered rock, both collected from nearby brackish environments to encourage them to construct nests. Nesting substrates collected from brackish environments were not included in freshwater boxes so as not to alter the salinity of the water, and because these substrates are not generally available in freshwater. All nesting substrates provided to the different males occur naturally in their source habitat and are distributed haphazardly within the same spawning areas in source lakes. After acclimatization for at least 24 hr in stock tanks, males were transferred to individual nesting boxes. Boxes were checked daily for signs of nest construction, and a nest was deemed complete when both an entry and exit hole were visible. The substrate on which males chose to build their nests, along with their ecotype, was recorded for each nest in order to investigate potential microhabitat differences in nest location between ecotypes. Males that failed to construct a nest within 7 days were replaced.

Following nest completion, a single heavily gravid female was introduced to each box in a small plastic jar (with the lid off), which subsequently acted as a refuge for the female during the trial (male courtship in sticklebacks can be aggressive, particularly when a larger male and a smaller female are involved). For trials involving females whose native salinity differed from that of the male (and therefore the water in the trial boxes), females were acclimatized to the same salinity as males over the 24‐hr period preceding the trial. Stickleback are naturally euryhaline and are capable of responding plastically, even to very abrupt changes in salinity (Taugbol et al., 2014), and therefore, this was unlikely to affect fish adversely during the trials. The behavior of both stickleback was recorded using a wide‐angle waterproof DB‐power digital video camera positioned at the opposite end of the box to the nest. Trials began upon first interaction between the male and female (which usually took place within 10 min of the female being introduced) and lasted for approximately 40 min. If mating had not taken place after this time, there is an extremely low likelihood of it ever occurring (Nagel & Schluter, 1998).

After trials were complete, females were removed from the boxes, anaesthetized, and killed according to Schedule One procedure. If spawning did not occur during the trial, females were stripped of their eggs to confirm readiness to spawn (eggs are easily removed from fully gravid females when gentle pressure is applied to the upper abdomen). Trials in which females could not easily be stripped of their eggs were discarded (this happened only seven times over 107 total trials). Females were measured for standard length because body size can be an important factor affecting mate choice in stickleback (Nagel & Schluter, 1998). In trials where eggs had been laid, the nest was removed from the male's box and eggs were carefully removed. Nests were subsequently returned to males, who were given 24 hr to rebuild their nests before they were offered to a subsequent female. Males of all ecotypes were used in multiple trials, 55 different males were used in total: 19 Obse anadromous, 8 Obse lagoon, 11 Faik anadromous, 6 Faik lagoon, and 11 Reiv freshwater (the individual male used was included as a random term in all statistical modeling to ensure differences between individual males did not affect the results). Each male was used in a maximum of three trials, separated by at least 24 hr. The order of trials was largely determined by the availability of females, and however, a male was never offered to a female from the same population twice. Once males had been used in up to three trials they were also anaesthetized and killed according to Schedule One, and measurements of standard length were taken.

### 2.3 Video analysis

Videos were visually analyzed using Behavioral Observation Research Interactive Software (BORIS) version 6.3.6 (Friard & Gamba, 2016). To assess differences in male courtship behavior between ecotypes, the number of times each male performed the following behaviors was recorded: zigzag dances (rapidly swimming from side to side toward a female), attacks (biting or bumping a female), charges (swimming rapidly toward a female), taps (tapping at the base of a female's tail when she is inside the nest), nest activities (adjusting the nest by fanning, or adding or removing substrate), leads (swimming toward the female fish then leading her to the nest), nest shows (showing the female the nest by probing the nest opening with his head), and dorsal pricks (pricking the female with dorsal spines). All of these behaviors are previously well described and can all form part of the courtship ritual in stickleback (Wilz, 1970; Wootton, 1984). The occurrence of spawning (females entering the nest and laying their eggs) was recorded for all trials. Although ecotypes are visually distinguishable by morphology, the observer had no prior knowledge of the different ecotypes or expectation of the outcome of the trials during video analysis. Trials in which the male and female failed to interact during the entirety of filming were discarded. Total trial times were recorded and if spawning occurred, trials ended once spawning were complete.

### 2.4 Statistical analysis

All statistical analyses were carried out in R version 3.5.2 (R Core Team, 2018). Where linear regression models were used, all numeric variables were centered and scaled prior to analysis, and model simplification was conducted starting with the fullest model and removing the interactions followed by least significant terms sequentially until reduced models were no longer an improvement on the most recent fuller model. The significance of terms in the model was assessed using likelihood ratio tests or *F* tests, as appropriate. The goodness‐of‐fit of the best fitting model was then evaluated using residual and Quantile‐Quantile (Q‐Q) plots, and models were transformed and re‐fitted if the necessary family criteria were violated.

To test whether species pair (lagoon and anadromous) males preferred different substrates for nest construction, we used a chi‐square test on the proportions of nest built on each of the five offered substrates by lagoon versus anadromous males. To test whether freshwater males preferred some nesting substrates over others, we performed a separate chi‐square test including only the freshwater males and the three nesting substrates which they were offered. Differences in the number of times male stickleback performed each courtship behavior were analyzed using zero‐inflated, negative binomial generalized linear mixed models (GLMMs) implemented using the glmmTMB R package (Brooks et al., 2017). The frequencies of each behavior were corrected to reflect differences in the length of trials when spawning occurred by dividing by the duration of the trial (in minutes). For all models of male behaviors, the individual male was fitted as a random effect to control for effects of males being used in multiple trials, and fitted predictor effects were as follows: male ecotype (anadromous, lagoon or freshwater), whether or not the male was displaying to a female of the same or a different ecotype (1 or 0) and the interaction between the two. To determine the contribution of each individual level (anadromous, lagoon, and freshwater) within the multi‐level predictor variable “male ecotype” when it had a statistically significant effect size in the optimal GLMM’s of male mating behaviors, post hoc estimated marginal means (EMM) were calculated for all pairwise level comparisons. *p*‐values were adjusted to account for multiple testing using the Tukey method for comparing families of three estimates. In the case of models where complete separation occurred (which resulted from lagoon males never performing some of the behaviors that were measured) post hoc EMM’s could not be calculated and therefore the contribution of the lagoon male effect to the significance of the male ecotype term was assessed in these cases by collapsing anadromous and freshwater males into a single level and comparing models on the two‐level and three‐level male ecotype variable using likelihood ratio tests based on the chi‐squared statistic.

To test for assortative mating and identify factors affecting spawning probability, we used a GLMM with a binomial error structure and logit link function, implemented using the lme4 package, version 1.1‐13 (Bates et al., 2015). The occurrence of spawning during the trials was used as a binary response variable, with year (2016 or 2017), absolute difference in body size (mm), female ecotype (freshwater, lagoon, or anadromous), whether both the male and female were of the same ecotype (0 or 1), and the interaction between the latter two as fixed effects in the model. To control for the effects of individual males being used in multiple trials, the individual male used in each trial was included as a random effect.

Our experiments were designed using anadromous and lagoon fish from two separate locations (lochs Obse and Faik), to test whether assortative mating between ecotypes would be maintained across populations. I.e. whether an anadromous female from one loch would prefer an anadromous male over a lagoon male, regardless of whether the male was from the same or a different loch. Therefore, we repeated the GLMM analysis on the occurrence of spawning using a reduced data set (*n* = 63 trials), which excluded all trials involving males or females from the allopatric population, Reiv, in order to test for the effects of loch in species pair trials. The model was specified as above, except the fixed effects were as follows: year (2016 or 2017), female ecotype (lagoon or anadromous), whether or not females and males were of the same ecotype (0 or 1) and whether or not females and males were from the same loch (0 or 1), and the interaction between female ecotype and loch.

## 3 RESULTS

A total of 91 successful mating trials (in which males and females interacted during the trial, and females either layed or were easily stripped of their eggs) were conducted (Table 2). Overall, spawning occurred in 24 of these 91 trials (26%). Spawning occurred in at least one trial for every possible combination of ecotypes apart from lagoon males with anadromous or freshwater females, although sample sizes for these combinations were low (Table 2). We found lagoon and freshwater males to have a low propensity to build nests in comparison with anadromous males and this coupled with variation in the availability of gravid females lead to variable sample sizes across trial combinations.

**TABLE 2:** Number of mate choice trials conducted for each combination of ecotypes

Male ecotype	Female ecotype		
	Anadromous	Lagoon	Freshwater
Anadromous	22	21	8
Lagoon	7	13	2
Freshwater	5	8	5

### 3.1 Nest location

**FIGURE 2:**
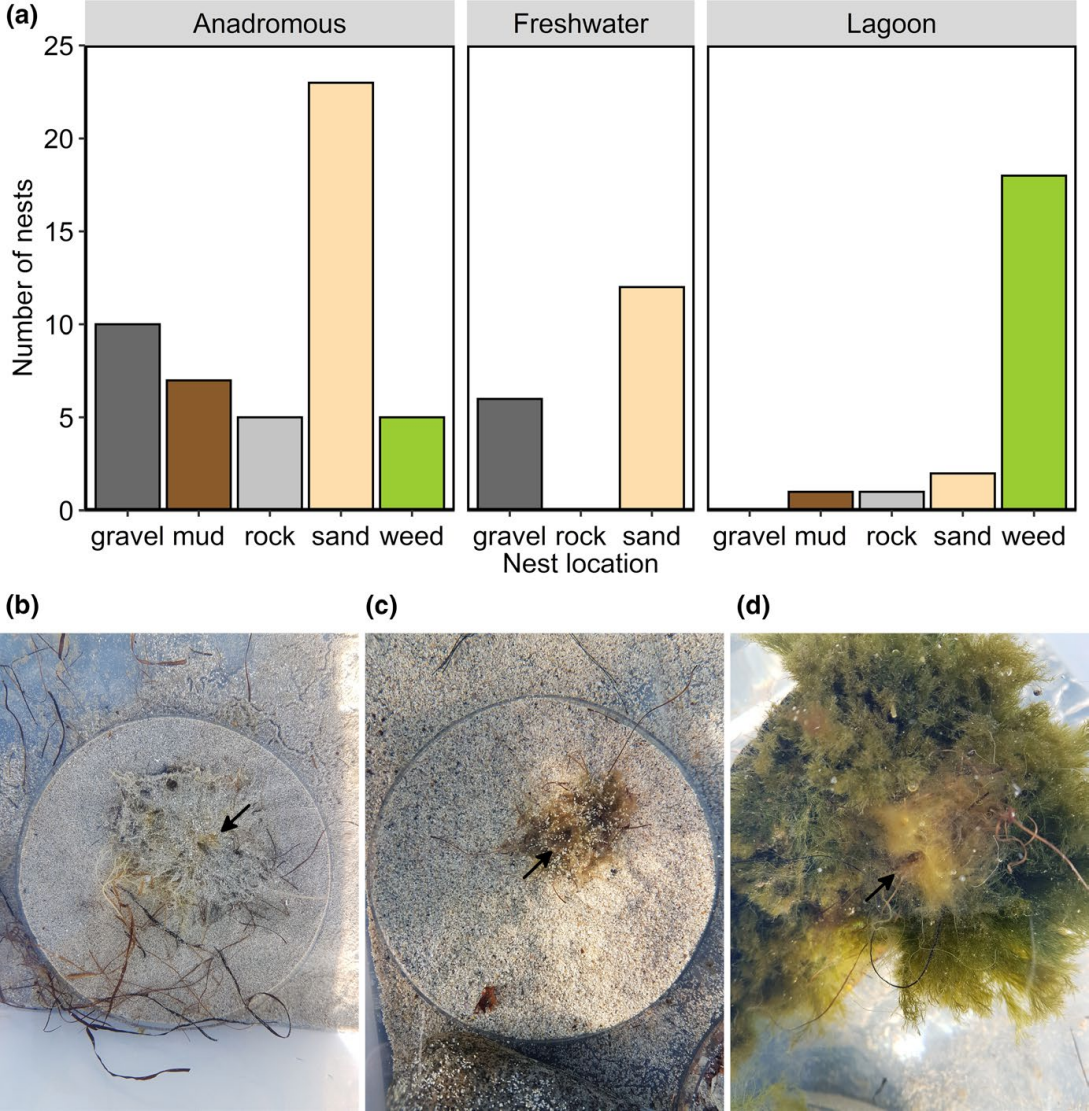
Nest locations. (a) Differences in the substrate on which males of different ecotypes chose to build their nests and examples of nests built on (b) mud (very fine particles), (c) sand (coarse particles), and (d) weed during mate choice trials. Arrows indicate nest entrances

Lagoon and anadromous males preferred to build their nests on different substrates (*χ* ^2^ = 32.03, *df* = 4, *p* < .0001, Figure 2a). Lagoon males showed an overwhelming preference for nesting on weed (82%), while anadromous males preferred other substrates in 90% of cases, particularly sand (46%), followed by gravel (20%). Freshwater males also showed a preference for some substrates over others (*χ* ^2^ = 12.00, *df* = 2, *p* = .0025, Figure 2a), choosing to nest on sand most frequently (67%), followed by gravel (33%), and never nesting on rock, Figure 2a. See Figure 2b–d, for examples, of nests on mud, sand, and weed, respectively.

### 3.2 Male courtship behavior

**FIGURE 3:**
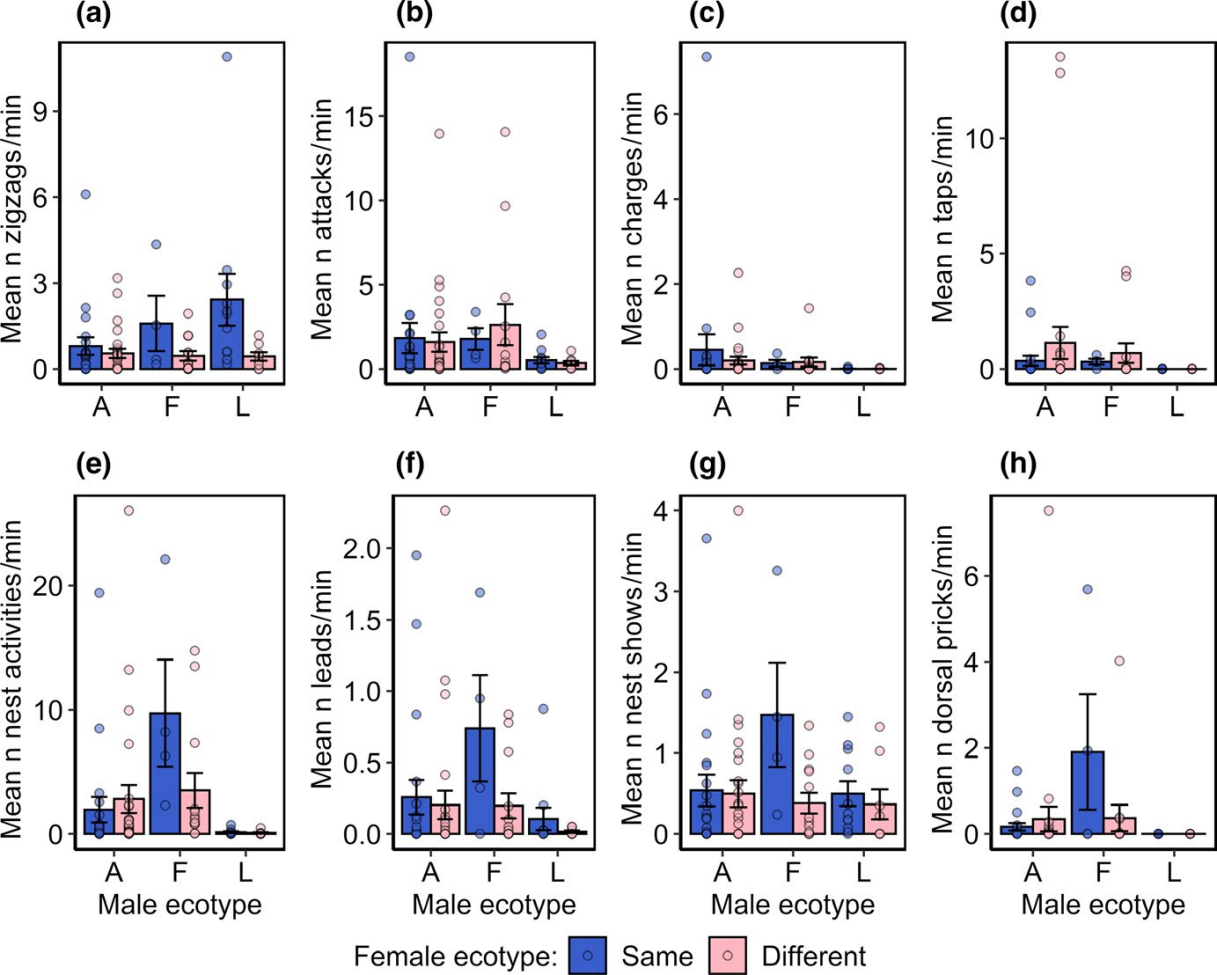
Variation in male courtship behavior. Graphs show differences in the mean occurrence per minute of (a) zigzags, (b) attacks, (c) charges, (d) taps, (e) nest activities, (f) leads, (g) nest shows, and (h) dorsal pricks performed by males of different ecotypes (A: anadromous, L: lagoon, F freshwater) toward either females of the same (blue bars) or different (pink bars) ecotypes as themselves. For all graphs, circles represent actual data points and error bars represent standard errors of each mean (*SEM*)

Male courtship behavior was recorded for 81 of the 91 successful mating trials. Males of different ecotypes differed in the types and quantities of courtship behaviors they performed (Figure 3a–h, Table 3). Male ecotypes differed in whether or not they performed attacks, charges, taps, nest activities, and dorsal pricking during courtship (Table 3). They also differed in the number of charges they performed toward females, the number of times they tapped females, the number of times they pricked females with their dorsal spines, and the number of times they performed nest tending activities during courtship (Table 3). These differences largely reflected a difference in the behavior of lagoon males, who were more likely not to perform behaviors or performed fewer behaviors than the other two ecotypes in all cases (see Appendix S1 and S2 for post hoc multi‐level factor comparisons). Freshwater and anadromous males did not differ from one another in any of the courtship behaviors we measured other than the number of nesting activities performed, with freshwater males performing more nesting activities than anadromous males (Figure 3a–h, Appendix S1 and S2).

**TABLE 3:** Differences in male mating behaviors

Male behavior (response variable)	Predictor variable	*df*	LRT	*p*‐value	Random effect variance ± *SD*
N zigzags	m ecotype	2	1.3516	.5087	0.000 ± 0.000
	ecotype same	1	5.7607	**.0164**	0.000 ± 0.000
	m ecotype * ecotype same	2	1.9216	.3826	0.000 ± 0.000
Zigzag	m ecotype	2	5.9776	.2008	0.000 ± 0.000
	ecotype same	1	10.4620	**.0053**	0.000 ± 0.000
	m ecotype * ecotype same	2	6.7626	.1490	0.000 ± 0.000
N attacks	m ecotype	2	3.2742	.1945	1.662 ± 1.289
	ecotype same	1	0.7847	.3757	1.658 ± 1.288
	m ecotype * ecotype same	2	1.2183	.5438	1.814 ± 1.347
Attack	m ecotype	2	29.6900	**<.0000**	1.662 ± 1.289
	ecotype same	1	0.2546	.8805	1.658 ± 1.288
	m ecotype * ecotype same	2	0.1497	.9973	1.814 ± 1.347
N charges	m ecotype	2	9.8070	**.0074**	2.590 ± 1.609
	ecotype same	1	0.0176	.8945	2.522 ± 1.588
	m ecotype * ecotype same	2	0.1700	.9185	2.813 ± 1.677
Charge	m ecotype	2	16.4340	**.0025**	2.590 ± 1.609
	ecotype same	1	6.4355	.1243	2.522 ± 1.588
	m ecotype * ecotype same	2	2.9535	.5656	2.813 ± 1.677
N taps	m ecotype	2	7.9094	**.0192**	2.462 ± 1.569
	ecotype same	1	0.4551	.49991	2.587 ± 1.609
	m ecotype * ecotype same	2	0.1433	.9309	2.449 ± 1.565
Tap	m ecotype	2	13.5020	**.0091**	2.462 ± 1.569
	ecotype same	1	2.4149	.2990	2.587 ± 1.609
	m ecotype * ecotype same	2	2.3677	.6685	2.449 ± 1.565
N nest activities	m ecotype	2	21.3000	**<.0000**	1.113 ± 1.055
	ecotype same	1	0.4640	.4958	1.202 ± 1.096
	m ecotype * ecotype same	2	2.2041	.3322	1.199 ± 1.095
Nest activities	m ecotype	2	36.3750	**<.0000**	1.113 ± 1.055
	ecotype same	1	1.2960	.5230	1.202 ± 1.096
	m ecotype * ecotype same	2	1.2200	.8748	1.199 ± 1.095
N leads to nest	m ecotype	2	2.9983	.2233	0.923 ± 0.961
	ecotype same	1	1.6056	.2051	1.056 ± 1.028
	m ecotype * ecotype same	2	0.7511	.6869	0.000 ± 0.000
Leading	m ecotype	2	6.1384	.1890	0.923 ± 0.961
	ecotype same	1	2.0669	.3558	1.056 ± 1.028
	m ecotype * ecotype same	2	2.8156	.5891	0.000 ± 0.000
N shows of nest	m ecotype	2	1.3400	.5117	0.000 ± 0.000
	ecotype same	1	0.6766	.4108	0.000 ± 0.000
	m ecotype * ecotype same	2	1.119	.5715	0.000 ± 0.000
Nest showing	m ecotype	2	5.3119	.2568	0.000 ± 0.000
	ecotype same	1	4.7987	.0908	0.000 ± 0.000
	m ecotype * ecotype same	2	6.0156	.1980	0.000 ± 0.000
N dorsal pricks	m ecotype	2	12.8220	**.0016**	0.418 ± 0.647
	ecotype same	1	0.0190	.8901	0.650 ± 0.806
	m ecotype * ecotype same	2	0.9132	.6334	0.000 ± 0.000
Dorsal pricking	m ecotype	2	13.0080	**.0112**	0.418 ± 0.647
	ecotype same	1	0.3748	.8291	0.650 ± 0.806
	m ecotype * ecotype same	2	1.9696	.7414	0.000 ± 0.000

Note

Table shows zero‐inflated negative binomial GLMM results for differences in male mating behaviors between male ecotypes (m. ecotype), different behaviors toward females of different ecotypes (ecotype same) and differences between male ecotypes in their response to females of different ecotypes (m ecotype * ecotype same interaction). LRT values are reported for comparisons of models before and after the removal of each predictor variable, with interactions removed first followed by least significant terms. Models testing the number of times a behavior was observed when it occurred are shown with response variables beginning *N*. All response variables not beginning *N* refer to models testing the probability of a behavior being observed (zero or nonzero). *p*‐values < .05 are highlighted in bold.

Abbreviations: *df*, degrees of freedom; ecotype same, whether or not the male was courting a female of the same ecotype as himself; LRT, likelihood ratio test statistics; m ecotype, ecotype of the male used in mate choice trials; *N*, number of (times each courtship behavior was performed); *SD*, one standard deviation.

The only behavior that males performed differently, if they were courting a female of their own ecotype or not, was the number of zigzag dances, with all males performing more zigzag dances for females of their own ecotype (Figure 3a, Table 3). The interaction between male ecotype and whether or not males were offered females of the same ecotype as themselves was not significant in any of our models (Table 3), indicating that males of all ecotypes responded in the same way to females of the same and different ecotype as themselves.

### 3.3 Female assortative mating

**FIGURE 4:**
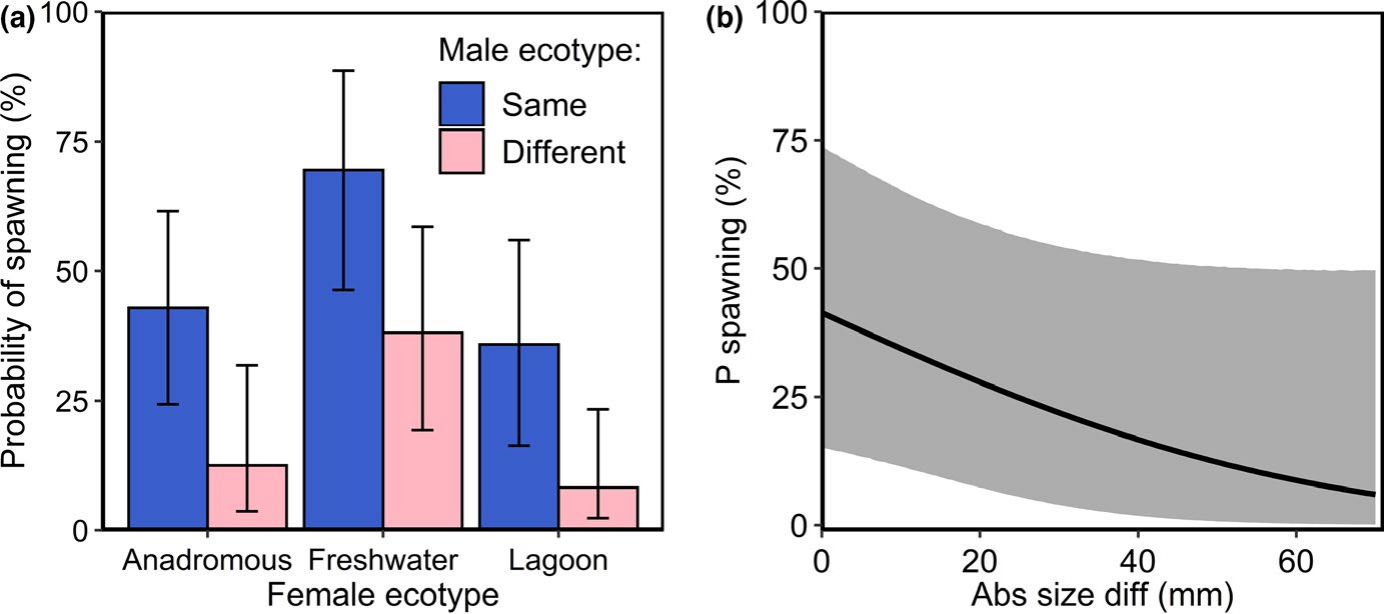
(a) Differences in spawning probability across ecotypes. Predicted probabilities of females of different ecotypes spawning with males of the same versus different ecotypes. Error bars show associated bootstrapped 95% confidence intervals. Predictions are based on a binomial generalized linear mixed model with individual male as a random effect and year, female ecotype and conspecific male as fixed effects. (b) Predicted probability of spawning (P spawning) with increasing differences in body size (Abs size diff) from a binomial generalized linear mixed model with year, absolute size difference, female ecotype, and whether or not male and female ecotypes were the same as predictor variables and individual male as a random effect. Bootstrapped 95% confidence intervals are indicated by the gray ribbon

Our results indicated that, overall, female stickleback exhibit positive assortative mating as spawning probability was higher when males were of the same ecotype as females (binomial GLMM, ecotype same: LR_1_ = 6.30, *p* = .0121, Figure 4a). This pattern remained consistent in the model that only included trials within species pairs, with females still being more likely to spawn with males of the same ecotype as themselves (binomial GLMM, ecotype same: LR_1_ = 20.02, *p* < .0001). In the species pairs, the female preference for males of their own ecotype occurred irrespective of whether males were from the same loch as the females (binomial GLMM, female ecotype x loch: LR_1_ = 0.03, *p* = .8700) and this effect was consistent across both lagoon and anadromous females (binomial GLMM, female ecotype: LR_1_ = 0.01, *p* = .9064).

The probability of spawning decreased with increasing body size differences between males and females, but this effect had large error margins and was not significant in our model (binomial GLMM, body size difference: LR_1_ = 1.24, *p* = .2648, Figure 4b).

The probability of spawning was considerably higher in 2016 than 2017 (binomial GLMM, year: LR_1_ = 16.12, *p* < .0001), and this effect was still present, although slightly weaker, in the species pairs alone (GLMM year: LR_1_ = 7.29, *p* = .007). The estimated variance component ± *SD* for the random effect of individual males was 1.27 ± 1.13 in the full model and 553.7 ± 23.53 in the species pair only model.

The strength of assortative mating did not differ between the three ecotypes (binomial GLMM, female ecotype x ecotype same: LR_2_ = 1.55, *p* = .4616, Figure 4a). There were, however, differences between ecotypes in the overall likelihood of females spawning, regardless of the ecotype of the male (binomial GLMM, female ecotype: LR_2_ = 7.03, *p* = .0297, Figure 4a). Re‐running the model with lagoon and anadromous females collapsed into a single level of the female ecotype factor (“saltwater females”) confirmed that these two ecotypes did not differ from each other in their overall spawning probability (Appendix S2), and therefore, the effect of female ecotype was caused by a greater probability of spawning in the allopatric freshwater ecotype, compared to the sympatric lagoon and anadromous ecotypes (Figure 4a).

## 4 DISCUSSION

We investigated behavioral differences during courtship and the presence and strength of assortative mating in newly described saltwater stickleback species pairs. We showed that species pair males differ in their nesting microhabitat preferences and courtship behavior and males of all ecotypes preferentially court females of the same ecotype as themselves. We also identified positive assortative mating in both species pair and allopatric females, but we did not find any differences in the strength of assortative mating between species pair and allopatric females, suggesting that female mating preferences have arisen as a by‐product of other ecological adaptations and are not necessarily under strong selection in species pairs. Finally, we found that fish being of the same ecotype was a much better predictor of spawning probability than difference in body size, implying that other characteristics that are divergent between ecotypes must be responsible for assortative mating. Our results indicate that divergent behavior and fine‐scale segregation of ecotypes when nesting (driven by divergent male nest substrate preferences) may drive assortative mating and play an important role in maintaining reproductive isolation in species pairs in the wild.

### 4.1 Differences in courtship behavior and nesting location

Courtship behavioral differences often play a key role in maintaining adaptive divergence in stickleback, and this has been particularly well studied in divergent benthic–limnetic (Foster, 1995; Foster et al., 2008; Shaw et al., 2007), lake–stream (Delcourt et al., 2008; Raeymaekers et al., 2010) and japan sea anadromous—pacific ocean anadromous ecotypes (Kitano et al., 2007). Lagoon stickleback have only recently been recognized as an ecotype in their own right (Dean et al., 2019; Ravinet et al., 2015), and so their courtship behavior has not previously been investigated. We showed that lagoon males performed fewer of many of the classic stickleback courtship behaviors than males of the other two ecotypes (or sometimes none). This means that sympatric lagoon and anadromous males differ markedly in courtship behavior, which may be involved in maintaining reproductive isolation in species pairs, just as in many other stickleback ecotypes. Anadromous stickleback are generally more aggressive than freshwater stickleback (McKinnon et al., 2012) and reduced courtship behavior has also been recorded in cannibalistic anadromous populations, in which large shoals of stickleback form, and attack and consume fry and/or eggs in the nests of lone males (Foster, 1995; Shaw et al., 2007). Lagoon males performed fewer (or none) of all courtship behaviors measured except for the number of zigzags, where they performed a higher frequency of zigzags than the other two ecotypes. Perhaps in a naturally sympatric setting lagoon males experience a trade‐off between the risks of being conspicuous during courtship and the benefits of attracting a female; avoiding most types of obvious courtship displays to reduce aggression and possible nest raids from anadromous counterparts.

We found that lagoon males also differed from the other two ecotypes in terms of their preferred nesting substrate, favouring weed over the sand or gravel utilized by freshwater and anadromous males. This is indicative of fine‐scale spatial structure in nesting location in species pairs, which could also be important for reproductive isolation (Borzee et al., 2016; Hagen, 1967; Pegoraro et al., 2016; Snowberg & Bolnick, 2012), as is the case in some benthic–limnetic species pairs (Ridgway & McPhail, 1987). Lagoon males are permanently resident in the sympatric lagoon spawning habitat, so theoretically have the opportunity to establish the most favorable spawning territories before anadromous males arrive. However, lagoon males are unlikely to be able to compete with the larger, more aggressive anadromous males to retain such territories. Nesting on weed may thus allow lagoon males to avoid territorial conflict with anadromous males if the preferred nesting substrate of anadromous males is sand or gravel. It could also confer an advantage to lagoon males if cannibalism or nest destruction by anadromous males occurs in species pairs, since nests suspended on weed are less conspicuous and probably less easy to attack than those built on other, harder substrates such as sand or gravel (Hagen, 1967; Kynard, 1979). It is probable that lagoon females share the preference of lagoon males for nests built on weed rather than other substrates as our data on spawning probability showed that lagoon females preferred lagoon males (with nests mostly on weed), rather than anadromous or freshwater males (with nests on sand/gravel).

Lackey and Boughman (2017) recently showed that divergence in habitat and mate choice is the two most important premating barriers during speciation in stickleback. They found that these two barriers evolve early and remain strong throughout the speciation process. We identified differences both in nesting microhabitat and in courtship behavior which strongly support the findings of Lackey and Boughman (2017), suggesting that these premating barriers may be particularly important for maintaining reproductive isolation in lagoon – anadromous species pairs. It is important to distinguish pairs of ecotypes that are transiently sympatric (such as the lagoon–anadromous pairs studied here) from many of the other fully sympatric pairs that exist within the stickleback species complex (e.g., benthic–limnetic). Divergent male mating behavior appears to be involved in reproductive isolation in both continually and transiently sympatric pairs (McPhail & Hay, 1983; Shaw et al., 2007). Interestingly, in freshwater–anadromous pairs (also transiently sympatric) freshwater males also zigzag more and bite less than anadromous males (McPhail & Hay, 1983), suggesting similar divergent mating behaviors may evolve repeatedly in transiently isolated species pairs.

Anadromous and freshwater males did not differ in any of the courtship behaviors other than their propensity to perform nest tending activities (which was only marginally significant, Appendix S1) or preferences for different nesting substrates in our experiments (although it is not possible to know whether freshwater males would have preferred nesting on weed over sand or gravel as low growing weed is not common in the lagoon from which we collected freshwater fish so they were not offered weed as a nesting substrate). Anadromous males approximate the marine ancestor of all freshwater and lagoon ecotypes (Colosimo et al., 2005) and this, coupled with the fact that anadromous behavior was very similar to that of freshwater fish, suggests the differences exhibited by lagoon males are probably derived traits, which may have evolved as a result of sympatry in species pairs.

Studies of mating preferences and prezygotic reproductive isolation primarily focus on female mate choice (Gavrilets et al., 2001; Gerhardt, 1994; Head et al., 2013; Laloi et al., 2011), but the potential for male mating preferences to be important is becoming increasingly appreciated (Hughes et al., 2013; McKinnon et al., 2012). We showed that males of all ecotypes preferentially courted females of the same ecotype as themselves, performing more zigzag dances toward females of the same ecotype. Zigzag dances involve rapid darts from side to side and are thus are probably the most energetically expensive of all courtship behaviors measured here, indicating that males make an active choice to direct costly mating behaviors toward females of their own ecotype. This could explain the increased probability for spawning between fish of the same ecotype and likely contributes to reproductive isolation in lagoon–anadromous species pairs. It also adds to the growing body of evidence to suggest that mate choice in stickleback is mutual (Kozak et al., 2009), and this is true across different species pairs. Although male mate choice was not a focus here, our findings identify the role of male choice in this species pair as a topic for future work.

### 4.2 Evidence for isolation based on body size

Size assortative mating is commonly involved in reproductive isolation in sympatric fish species (Foote & Larkin, 1988; McKaye, 1986; McKinnon et al., 2004; Rueger et al., 2016; Sigurjonsdottir & Gunnarsson, 1989) and is well described in stickleback (Boughman et al., 2005; Conte & Schluter, 2013; McKinnon et al., 2004, 2012; Nagel & Schluter, 1998). However, we found only weak (nonsignificant) evidence that assortative mating was related to body size, which was somewhat unexpected. One possible explanation for this is that body size is utilized by stickleback to discriminate between ecotypes during courtship in saltwater species pairs, but not to select mates within ecotypes. Mate choice experiments involving lava and nitella stickleback ecotypes in Iceland identified a similar scenario, with no evidence for a role for size assortative mating between ecotypes, although there was a suggestion that both lava and nitella females preferred larger males (Olafsdottir et al., 2006). Our findings, therefore, add to evidence which suggests that factors affecting mate choice between ecotypes are not necessarily replicated across independent stickleback species pairs. Interestingly, in lava and nitella pairs where there was no evidence for size assortative mating, the two morphs differed in nesting location and structure (Olafsdottir et al., 2006), which is exactly the pattern we identified in our data. This suggests that there may be a consistent pattern in stickleback in which differences in male nesting behavior, particularly in nesting location may be particularly important for between—ecotype mate choice when variation in body size does not play a significant role. It may also be that other factors that were not measured in our experiment, such as nuptial color, are more important in driving assortative mating, but further experiments would be necessary to determine their effects. This provides an interesting avenue for future research.

### 4.3 By‐product versus adaptive assortative mating

Positive assortative mating is extremely common in taxa composed of multiple ecotypes (Hollander et al., 2005; Jarvis et al., 2017; Machado‐Schiaffino et al., 2017) and is well described in stickleback (Conte & Schluter, 2013; Ingram et al., 2015; McKinnon & Rundle, 2002), and therefore, it is not surprising that we identified it in all ecotypes in this study. The preferences we identified could have a genetic basis, but species pairs of benthic and limnetic stickleback acquire their ecotype specific preferences in a process of sexual imprinting on their fathers during early development (Kozak et al., 2011) (stickleback fry are cared for by males for a number of weeks after hatching), so it is also possible that imprinting plays a role here and the mechanism of heritability of mating preferences in Uist stickleback would be an interesting topic of further investigation.

Allopatric populations cannot hybridize, so by default, any assortative mating that exists between totally isolated populations can only have evolved as a by‐product of their isolation (Schluter, 2001). We detected assortative mating in both allopatric and sympatric stickleback populations, which illustrates that it has evolved at least partially as a by‐product of other adaptations in this system. Given the substantial phenotypic adaptations, including differences in body armor, size, and shape (Campbell, 1979; MacColl et al., 2013; Magalhaes et al., 2016), across stickleback populations on North Uist, some by‐product assortative mating would be expected (Dodd, 1989; Kilias et al., 1980). This mechanism is also implicated in causing assortative mating in other reproductively isolated, allopatric stickleback populations (Vines & Schluter, 2006), which suggests that reasonably substantial assortative mating, that is purely a by‐product of other differences, may be common among stickleback. We did not detect any exaggeration of assortative mating in species pairs compared with naïve allopatric stickleback, which suggests that if a signal of selection on assortative mating is present in species pairs it is not strong enough to be detected in our experiments. It is also possible that the assortative mating that we detected in species pairs arose via other nonadaptive, but population‐specific mechanisms such as genetic drift or sexual selection.

## 5 CONCLUSIONS

We identified behavioral differences, both in courtship rituals and preferred nesting substrate, which likely contribute to maintaining reproductive isolation in newly described saltwater stickleback species pairs. We found evidence that both species pair and allopatric males selectively court females of the same ecotype as themselves. We also showed that females in species pairs prefer to mate with males of their own ecotype, but these preferences are not driven by the commonly implicated magic trait, body size, and are also no stronger in sympatric species pairs versus allopatric populations. Our results suggest that divergent behavior, particularly that which causes fine‐scale differences in nesting location, is probably important for maintaining reproductive isolation in sympatric populations. Our findings also highlight that reasonably strong assortative mating can develop as a by‐product of other differences between populations, and strong selection on mate choice may not be particularly important in this system.

## CONFLICT OF INTEREST

The authors declare no competing interests.

## AUTHOR CONTRIBUTIONS

**Laura L. Dean:** Conceptualization (equal); Formal analysis (equal); Investigation (equal); Methodology (equal); Writing‐original draft (lead); Writing‐review & editing (equal). **Hannah R. Dunstan:** Formal analysis (supporting). **Amelia Reddish:** Investigation (equal). **Andrew D. C. MacColl:** Conceptualization (equal); Data curation (equal); Formal analysis (supporting); Funding acquisition (lead); Investigation (equal); Methodology (equal); Project administration (lead); Resources (lead); Supervision (lead); Writing‐review & editing (equal).

## Supporting information

Appendix S1‐S2

## Data Availability

All data from this manuscript are accessible in the Dryad digital repository (https://doi.org/10.5061/dryad.xd2547dg0).
