# Supplementary material for: Courtship behavior, nesting microhabitat, and assortative mating in sympatric stickleback species pairs
Source: Ecol Evol. 2021 Jan 29;11(4):1741–55. doi: 10.1002/ece3.7164 (PMC7882950; doi:10.1002/ece3.7164)
Supplement: Supplementary file 1 — Appendix S1‐S2 [file ECE3-11-1741-s001.docx]

**Appendices**

**Appendix S1.** Post-hoc statistical comparisons. Estimated marginal means for multi-level factor comparisons of significant predictor variables in the optimal generalized linear-mixed effects models of male mating behaviours. Models testing the number of times a behaviour was observed when it occurred are shown with response variables beginning N. All response variables not beginning N refer to models testing the probability of a behaviour being observed (zero or non-zero). *P*-values are adjusted to account for multiple testing using the Tukey method for comparing families of three estimates and *P*-values < 0.05 are highlighted in bold.

| **Response variable** | **Predictor variable** | **Comparison** | **Response estimate ± SE** | ***df*** | ***t* ratio** | ***p*-value** |
| --- | --- | --- | --- | --- | --- | --- |
| Attack | m ecotype | anad : fresh | -763 ± 355.3 | 77 | -2.147 | 0.0806 |
|  |  | anad : lagoon | 126 ± 42.9 | 77 | 2.930 | **0.0095** |
|  |  | fresh: lagoon | 888 ± 353.4 | 77 | 2.514 | **0.0320** |
| N charges | m ecotype | anad : fresh | -1.080 ± 1.130 | 77 | -0.954 | 0.6079 |
|  |  | anad : lagoon | 3.890 ± 1.350 | 77 | 2.870 | **0.0145** |
|  |  | fresh: lagoon | 4.960 ± 1.560 | 77 | 3.187 | **0.0058** |
| Charging | m ecotype | anad : fresh | -64.2 ± 61.1 | 77 | -1.050 | 0.5450 |
|  |  | anad : lagoon | 30.1 ± 13.5 | 77 | 2.625 | **0.0470** |
|  |  | fresh: lagoon | 94.3 ± 59.6 | 77 | 1.583 | 0.2531 |
| N nest activities | m ecotype | anad : fresh | -1.620 ± 0.663 | 77 | -2.438 | **0.0445** |
|  |  | anad : lagoon | 2.940 ± 0.722 | 77 | 4.070 | **0.0003** |
|  |  | fresh : lagoon | 4.560 ± 0.850 | 77 | 5.361 | **<0.0001** |
| Nest activities | m ecotype | anad : fresh | -763 ± 390.0 | 77 | -1.955 | 0.1235 |
|  |  | anad : lagoon | 233 ± 61.6 | 77 | 3.778 | **0.0005** |
|  |  | fresh : lagoon | 995 ± 385.2 | 77 | 2.584 | **0.0264** |

N: number of, anad: anadromous, fresh: freshwater, SE: standard error, *df*: degrees of freedom

**Appendix S2.** Level reduction model comparisons for statistically significant multi-level factors in generalized linear mixed effect models (GLMMs). Models testing the number of times a behaviour was observed when it occurred are shown with response variables beginning N. All response variables not beginning N refer to models testing the probability of a behaviour being observed (zero or non-zero).

| **Response variable** | **Factor levels in compared models** | ***df*** | **χ^2^** | ***p*-value** |
| --- | --- | --- | --- | --- |
| N taps | m ecotype (anadromous, lagoon, freshwater)  vs.  m ecotype (lagoon, other) | 1 | 1.3139 | 0.2517 |
| Tapping | m ecotype (anadromous, lagoon, freshwater)  vs.  m ecotype (lagoon, other) | 1 | 3.2705 | 0.1949 |
| N dorsal pricks | m ecotype (anadromous, lagoon, freshwater)  vs.  m ecotype (lagoon, other) | 1 | 0.0000 | 1.0000 |
| Dorsal pricking | m ecotype (anadromous, lagoon, freshwater)  vs.  m ecotype (lagoon, other) | 1 | 1.3670 | 0.5049 |
| P spawning | f ecotype (anadromous, lagoon, freshwater)  vs.  f ecotype (freshwater, other) | 1 | 0.3324 | 0.5643 |

N: number of, P: probability of, m: male, f: female, *df*: degrees of freedom.
